# Effects of Radiotherapy or Radical Prostatectomy on the Risk of Long-Term Heart-Specific Death in Patients With Prostate Cancer

**DOI:** 10.3389/fonc.2020.592746

**Published:** 2020-11-17

**Authors:** Yadong Guo, Xiaohui Dong, Fuhan Yang, Yang Yu, Ruiliang Wang, Aimaitiaji Kadier, Wentao Zhang, Shiyu Mao, Aihong Zhang, Xudong Yao

**Affiliations:** ^1^Department of Urology, Shanghai Tenth People's Hospital, Tongji University, Shanghai, China; ^2^Department of Geriatrics, Shanghai General Hospital, Shanghai Jiao Tong University School of Medicine, Shanghai, China; ^3^Department of Medical Statistics, Tongji University School of Medicine, Shanghai, China

**Keywords:** prostate cancer, radiotherapy, radical prostatectomy, cardiovascular disease, competing risk, SEER

## Abstract

**Objective:** The prognosis of patients with prostate cancer (PCa) has improved in recent years, but treatment-related cardiotoxicity remains unclear. This study investigated the heart-specific mortality and prognostic factors of patients with PCa after radiotherapy (RT) or radical prostatectomy (RP), and compared their long-term heart-specific mortality with that of the general male population.

**Materials and Methods:** Data were taken from the Surveillance, Epidemiology, and End Result (SEER) database. Patients with PCa were included who underwent RT or RP from 2000 to 2012, and were followed through 2015. A cumulative mortality curve and a competitive risk regression model were applied to assess the prognostic factors of heart-specific mortality. Standardized mortality rates (SMRs) were calculated.

**Results:** Of 389,962 men, 49.7% and 50.3% received RP and RT, respectively. The median follow-up was 8.3 years. For patients given RT, in about 9 years postdiagnosis, the cumulative mortality due to heart-specific disease exceeded that due to PCa. In patients who underwent RP, cumulative mortality from heart-specific disease or PCa was comparable. Relative to the general male population, overall, the heart-specific mortality of patients with PCa receiving RT or RP was not higher, but in patients aged 70 to 79 years, those given RT experienced slightly higher heart-specific mortality than the age-matched general population.

**Conclusions:** Patients with PCa treated with RT or RP overall do not incur risk of heart-specific mortality higher than that of the general male population, except for patients aged 70–74 years receiving RT.

## Introduction

Prostate cancer (PCa) is the most common primary malignant tumor and the second leading cause of cancer deaths in the United States, where the number of new cases and deaths in 2020 is estimated at 191,930 and 33,330, respectively (1). The incidence and mortality of PCa have decreased over the last three decades, but the proportion of noncancer causes of death in this population is still high, especially those related to cardiovascular disease (2–4).

Advancements in the treatment of PCa include various surgical treatments, hormone therapy, and radiotherapy (RT), but while PCa survival rates have significantly improved (5–7), deaths due to related diseases or comorbidities have increased (8). Indeed, patients with some treated cancers have a higher risk of death due to other causes. In particular, for specific cancers after RT, death rates associated with cardiovascular disease are significantly higher (9–11). While aging is an important natural driver of cardiovascular incidence and mortality in the general population, the mortality of cardiovascular disease in cancer patients is two- to sixfold that of the general population (12, 13). There is a question whether the cancer treatments themselves may contribute to the risk of cardiovascular disease.

Very few studies have compared the heart-specific death rates of patients with PCa with that of the general population (14, 15). Such comparisons are essential to reveal the potential cardiotoxic effects of PCa treatments.

The present study compared the long-term heart-specific mortality of men with PCa who underwent RT or radical prostatectomy (RP), with that of the general male population of the United States. The data was obtained from the SEER (Surveillance, Epidemiology, and End Result) database.

## Methods

### Data Sources

The SEER database comprises data from 18 regional cancer registration centers in the United States and accounts for ~34.6% of the population. The database collects comprehensive demographic and cancer-specific information, including the causes of death as coded by the International Classification of Diseases 10 (ICD-10; Supplementary Table 1).

### Patient Selection

The present analysis was limited to men aged ≥15 years, who received a diagnosis of primary malignant PCa from January 1, 2000 to December 31, 2012. Patients with unknown treatments, unknown survival outcomes, and missing death information were excluded. The end of the follow-up period was December 31, 2015.

The following data were collected: age at diagnosis, year of diagnosis, marital status, and ethnicity; histopathological type of tumor, cancer stage, tumor grade, and treatment (RP or RT). Patients were stratified by treatment (RP or RT), the age at diagnosis (<50, 50–64, 65–74, or ≥75 years), and the year of diagnosis (year 2000–2005 or 2006–2012). The categories of marital status included married, widowed or divorced, single, and unknown. Ethnicity was recorded as Caucasian, African-American, other, and unknown. Histopathological type included adenocarcinoma and other. The PCa stage was recorded as local, regional, distant, or unknown; and tumor grade as I through IV or unknown.

Finally, overall, 389,962 patients met the inclusion criteria. Among them, there were 196,009 (50.3%) and 193,953 (49.7%) patients in the RP and RT groups, respectively.

### Statistical Analysis

The baseline characteristic distribution of the RP and RT groups was described by composition ratio, and the comparison of two or more composition ratios was by chi-squared test. A competitive risk model was used to estimate PCa-specific crude cumulative mortality and heart crude cumulative mortality of patients with PCa and to plot the crude cumulative mortality curve, further stratified by age. The Fine-Gray competitive risk model was applied to adjust for the confounding effects of age at diagnosis, year of diagnosis, ethnicity, tumor histopathological type, cancer stage, and tumor grade, and to evaluate the risk of PCa-specific death and heart-specific death in patients undergoing RT or RP. Cox proportional hazards regression was used to assess the risk of all-cause mortality.

Standardized mortality ratios (SMRs) were used to compare the all-cause mortality and heart-specific mortality of patients with PCa with that of the general male population, stratified according to age and stage. The SMR for a specific cause is the ratio of the total number of observed deaths to the number expected from the age-specific reference. The expected number of deaths was determined by multiplying the cumulative age of patients with PCa in 5-year-increment age groups (50–54, 55–59, 60–64, 65–69, 70–74, 75–79, 80–84, and >85 years) with the age-specific mortality of the general male population during 2012–2014.

The age-specific mortality of the general male population in the United States was obtained from the Centers for Disease Control and Prevention (CDC) WONDER Mortality Underlying Cause of Death online database (16). The SMR and 95% confidence interval were calculated for the different treatments, for all-cause mortality, and for heart-specific mortality. Statistical significance was defined by a two-sided *P* < 0.05. Statistical analyses were performed with Stata/MP 14.0 and R software package.

## Results

### Patient Characteristics

The study population comprised 389,962 patients with PCa who fulfilled the inclusion criteria as identified from the SEER database. Of these, 49.7% received RP, and 50.3% received RT. The median follow-up times of the RT and RP groups were, respectively, 98 months [interquartile range (IQR) 65–138 months] and 93 months (IQR 59–131 months).

The age groups <50, 50–64, 65–74, and ≥ 75 years made up 4.1, 37.1, 11.4, and 47.45% of the population, respectively (Table 1). For men aged <50 and ≥75 years, the majority were administered RP (80.7 and 65.3%, respectively). However, men aged 50–64 and 65–74 years were more likely to have undergone RT (60.8 and 92.1%). The total population was 79.5, 14.4, and 6.1%, Caucasian, African-American, and other/unknown ethnicities, respectively. In the Caucasian, African-American, and other/unknown cohorts, 48.8, 56.5, and 55.3% received RT, while 51.2, 43.5, and 44.7% were administered RP.

**Table 1:** Basic characteristics at diagnosis of patients with prostate cancer (PCa) in the prostatectomy (RP) and radiotherapy (RT) treatment groups^*^.

		**RT**	**RP**	***P***
Subjects, *n*		196,009	193,953	
Age, years		60.52 ± 7.20	67.55 ± 8.19	<0.001
Age (%)	<50	3,103 (1.6)	12,978 (6.7)	<0.001
	50–64	87,903 (44.8)	56,658 (29.2)	
	65–74	40,789 (20.8)	3,477 (1.8)	
	≥75	64,214 (32.8)	120,840 (62.3)	
Year of diagnosis	2000–2005	88,567 (45.2)	77,153 (39.8)	<0.001
	2006–2012	107,442 (54.8)	116,800 (60.2)	
Ethnicity	White	151,223 (77.2)	158,923 (81.9)	<0.001
	Black	31,648 (16.1)	24,397 (12.6)	
	Other	10,550 (5.4)	9,002 (4.6)	
	Unknown	2,588 (1.3)	1,631 (0.8)	
Histologic subtype	Adenocarcinoma	192,440 (98.2)	188,175 (97.0)	<0.001
	Other	3,569 (1.8)	5,778 (3.0)	
Grading	I	2,286 (1.2)	1,707 (0.9)	<0.001
	II	103,577 (52.8)	91,387 (47.1)	
	III	83,304 (42.5)	98,825 (51.0)	
	IV	528 (0.3)	410 (0.2)	
	Unknown	6,314 (3.2)	1,624 (0.8)	
Stage	Local	180,009 (91.8)	135,411 (69.8)	<0.001
	Regional	6,767 (3.5)	57,774 (29.8)	
	Distant	6,733 (3.4)	361 (0.2)	
	Unknown	2,500 (1.3)	407 (0.2)	
Cause of death	Alive	144,588 (74.5)	178,434 (91.0)	<0.001
	Prostate cancer	12,786 (6.6)	3,484 (1.8)	
	Heart-specific disease	13,688 (7.1)	3,825 (1.9)	
	Other	24,947 (12.7)	8,210 (4.2)	

* *Reported as n (%), unless noted otherwise. P-values refer to comparisons between the RT and RP populations for the clinicopathological characteristic*.

Histologic subtypes were recorded as adenocarcinoma (97.6%) and other (2.4%) (Table 1). Of patients with adenocarcinoma, 50.6 and 49.4% were treated with RT and RP. Patients with histologic subtypes other than adenocarcinoma were more likely to be given RP (61.8%) than RT (38.2%). Most of the patients (96.7%) had tumor grades II or III. Of these, 49.6 and 50.4% received RT and RP. Patients with tumor grades I and IV received RT (57.2, 56.3%) and RP (42.7, 43.7%).

Of the overall population, cancer stages at diagnosis were local (80.9%), regional (16.5%), distant (1.8%), and unknown (0.7%) (Table 1). Of patients with local disease, 57.1% were given RT, and 42.9% received RP. Patients with regional disease were much more likely to be given RP (89.5%) than RT (10.5%), while patients with distant disease were much more likely to undergo RT (94.9%) than RP (5.1%).

At the last follow-up, 82.8% of the population was living, while the remainder (17.2%) had suffered mortality due to PCa (4.2%), heart-specific disease (4.5%), or other causes (8.5%) (Table 1). Of those who were living, 55.2% had undergone RP, and 44.8% had been given RT. Of patients who died, a greater percentage had been given RT (73.7%) rather than RP (26.3%).

Specifically, of patients who died of PCa, heart-related disease, or other causes, only 21.4, 21.8, and 31.0% received RP, respectively, while the majorities (78.6, 78.2, and 69.0%) had received RT.

### Cumulative Mortality

**Figure 1:**
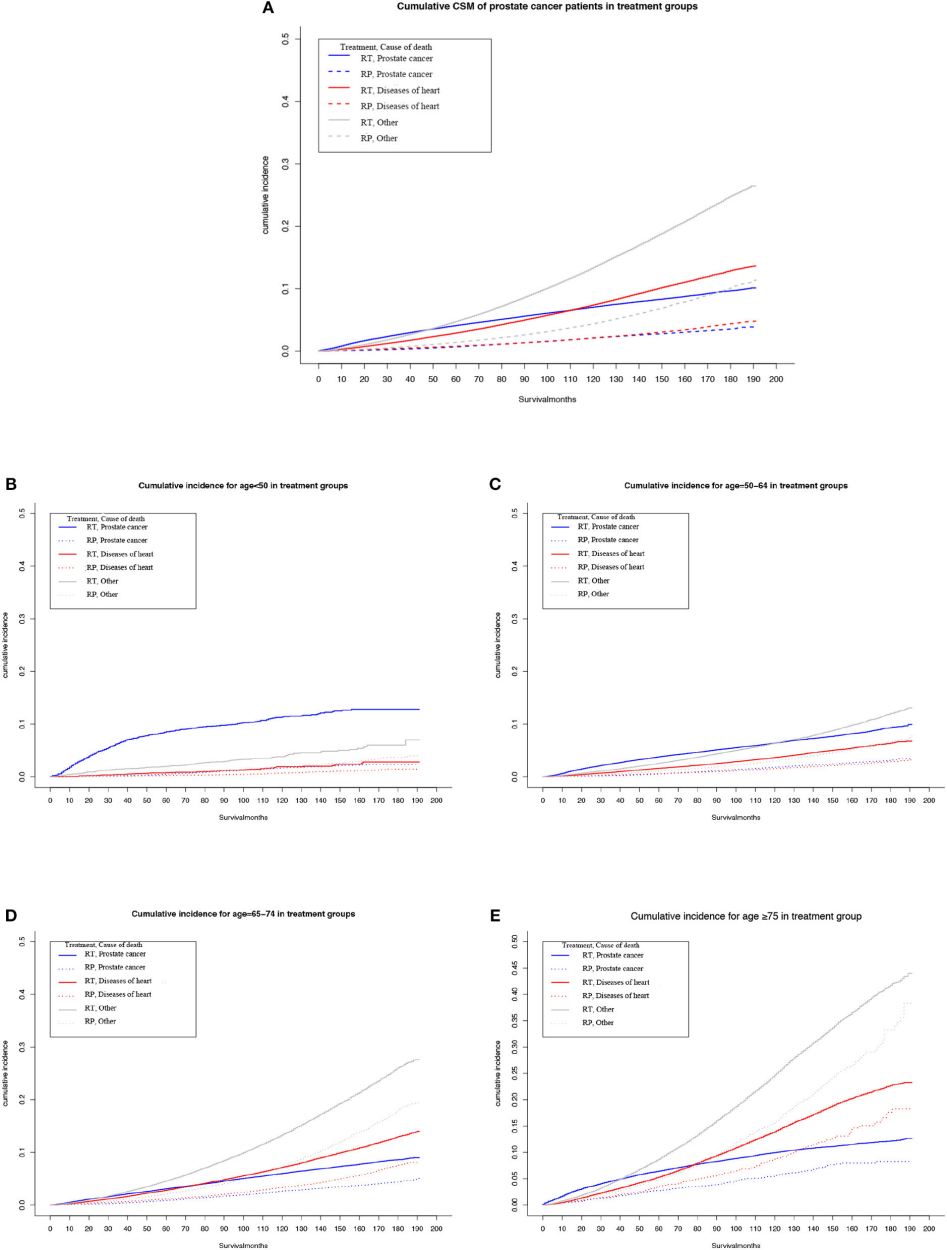
Multivariable adjusted cumulative incidence function curve of patients with prostate cancer (PCa) undergoing prostatectomy (RP) or radiotherapy (RT). **(A)** Cause-specific mortality of the overall PCa population. **(B–E)** Heart-specific and PCa mortality rates by age at diagnosis: **(B)** <50 years, **(C)** 50–64 years, **(D)** 65–74 years, and **(E)** ≥75 years.

Among the patients who were administered RT, the highest cumulative mortality was due to causes unrelated to heart disease or PCa, but ~110 months after diagnosis of PCa, the cumulative mortality due to heart-specific disease exceeded that due to PCa (Figure 1A).

Among patients who underwent RP, the cumulative mortality due to heart-specific disease was similar to the cumulative mortality due to PCa.

Subgroup analyses determined that, as age at diagnosis of PCa increased, or duration of follow-up, the cumulative heart disease-specific mortality also rose. For men who were <65 years old at the time of diagnosis and given RT, the highest cumulative mortality rate was for PCa (Figures 1B,C). However, for men who underwent RT and aged ≥65 years, at ~90 months after diagnosis, the cumulative mortality from heart-specific disease exceeded the cumulative mortality from PCa (Figures 1D,E).

For men who were <65 years old at the time of diagnosis and given RP, cumulative mortality from heart-specific disease was close to that due to PCa (Figures 1B,C). However, the cumulative mortality of heart-specific disease exceeded that due to PCa for the following age groups and follow-up times: 65–74 years old at ~90 months; and ≥75 years at ~60 months (Figures 1D,E).

### Mortality Compared With the General Population

The heart-specific and all-cause mortalities of patients with PCa undergoing RT or RP were standardized relative to the general male population, by age and cancer stage (Table 2). For men treated with RT, the heart-specific mortality of patients aged 70–74 years was slightly higher than that of the general population (SMR 1.21, 95% CI 1.06–1.38), but this was not true of any other age group, cancer stage, or in the study population overall. Overall, the death rate due to all causes in patients who received RT was 5% less than that of the general population (SMR 0.95, 95% CI, 0.93–0.97). However, patients aged 50–54 years who received RT had nearly a threefold higher death rate due to any cause, relative to the general male population (SMR 2.9, 95% CI 1.21–6.96), this higher rate decreased with each 5-year increment in age.

**Table 2:** Age-specific and overall standardized mortality ratios (SMRs) for the years 2012–2014 among patients with PCa who survived at least 10 years relative to the general male population of the United States^*^.

		**SMR heart-specific (95% CI)**		**SMR overall (95% CI)**	
		**RT**	**RP**	**RT**	**RP**
Age	50–54	Nil	0.76 (0.19–3.06)	**2.9 (1.21–6.96)**	0.99 (0.55–1.79)
	55–59	0.81 (0.26–2.51)	0.7 (0.4–1.23)	**1.63 (1.1–2.41)**	0.79 (0.61–1.03)
	60–64	1.23 (0.85–1.78)	0.45 (0.32–0.63)	**1.34 (1.12–1.6)**	0.65 (0.57–0.75)
	65–69	1.09 (0.88–1.36)	0.48 (0.39–0.59)	**1.16 (1.05–1.29)**	0.55 (0.5–0.61)
	70–74	**1.21 (1.06–1.38)**	0.51 (0.44–0.6)	**1.08 (1.01–1.16)**	0.56 (0.52–0.6)
	75–79	1.1 (1–1.21)	0.56 (0.5–0.64)	0.99 (0.94–1.04)	0.62 (0.59–0.66)
	80–85	0.97 (0.9–1.05)	0.62 (0.54–0.7)	0.94 (0.91–0.98)	0.66 (0.62–0.7)
	85+	0.91 (0.86–0.96)	0.54 (0.45–0.64)	0.89 (0.87–0.92)	0.58 (0.53–0.64)
Stage	Local	0.97 (0.94–1.01)	0.51 (0.47–0.56)	0.93 (0.91–0.95)	0.52 (0.5–0.54)
	Regional	1.13 (0.92–1.4)	0.64 (0.57–0.71)	**1.39 (1.26–1.54)**	0.81 (0.77–0.85)
	Distant	1.07 (0.59–1.94)	0.68 (0.1–4.8)	**1.87 (1.48–2.36)**	1.55 (0.81–2.98)
	Unknown	0.81 (0.56–1.17)	0.15 (0.02–1.05)	1.14 (0.97–1.34)	1.00 (0.68–1.46)
	Total	0.98 (0.94–1.01)	0.55 (0.51–0.59)	0.95 (0.93–0.97)	0.61 (0.59–0.63)

* *Bolded SMRs are significantly different from 1.00 (P < 0.05)*.

* *The SMR, 95% CI, and P value were not calculated*.

Among the patients who underwent RP, the rates of both heart-specific and all-cause deaths were not significantly higher than that of the general population, neither in the overall population nor in any subcategory (Table 2).

### Relative Risk Model for Specific Causes

The Fine–Gray model of competitive risk is used to examine the associations between prognostic factors and heart disease mortality or PCa-specific mortality in patients receiving RT or RP (Table 3). With each 5-year advance in age, the risk of heart-specific death gradually increased from 0.44 (beginning age 50 years) to 4.38 (older than 75 years) (Table 3). In addition, patients who received a diagnosis of PCa between 2006 and 2012 had a significantly lower risk of heart-specific death, relative to those with diagnoses in the years 2000 to 2005 [subdistribution hazard ratio (SHR), 0.67; 95% CI, 0.65–0.70].

**Table 3:** All-cause, heart-related, and prostate-related mortality of patients by demographic and clinical characteristics at diagnosis.

**Variables**	**All cause**		**Heart disease**		**PCa**	
	**HR^**a**^**	***P***	**SHR^**b**^**	***P***	**SHR^**b**^**	***P***
**Age**						
<50	0.71 (0.66,0.76)	<0.001	0.44 (0.37,0.52)	<0.001	1.02 (0.92,1.13)	0.658
50–64	1.00^c^		1.00^c^		1.00^c^	
65–74	1.93 (1.89,1.96)	<0.001	2.15 (2.06,2.23)	<0.001	1.07 (1.02,1.11)	<0.001
≥75	3.87 (3.78,3.96)	<0.001	4.38 (4.19,4.59)	<0.001	1.31 (1.25,1.38)	<0.001
**Year of diagnosis**						
2000–2005	1.00^c^		1.00^c^		1.00^c^	
2006–2012	0.79 (0.78–0.81)	<0.001	0.67 (0.65,0.70)	<0.001	0.63 (0.61,0.65)	<0.001
**Race**						
White	1.00^c^		1.00^c^		1.00^c^	
Black	1.29 (1.26,1.32)	<0.001	1.42 (1.37,1.48)	<0.001	1.05 (1.01,1.11)	0.029
Other	0.77 (0.74,0.80)	<0.001	0.73 (0.68,0.79)	<0.001	0.74 (0.69,0.81)	<0.001
Unknown	0.23 (0.19,0.27)	<0.001	0.14 (0.09,0.23)	<0.001	0.11 (0.06,0.18)	<0.001
**Histologic subtype**						
Adenocarcinoma	1.00^c^		1.00^c^		1.00^c^	
Other	1.18 (1.13,1.23)	<0.001	0.89 (0.80,0.98)	0.023	1.30 (1.25–1.34)	<0.001
**Grading**						
I	1.00^c^		1.00^c^		1.00^c^	
II	0.96 (0.89,1.03)	0.226	0.90 (0.80,1.01)	0.079	1.01 (0.81,1.25)	0.962
III	1.40 (1.31,1.51)	<0.001	1.00 (0.88,1.13)	0.961	3.14 (2.53,3.89)	<0.001
IV	2.37 (2.09,2.69)	<0.001	0.94 (0.68,1.29)	0.708	5.49 (4.14,7.27)	<0.001
Unknown	1.66 (1.54,1.80)	<0.001	0.95 (0.81,1.11)	0.498	3.43 (2.74,4.30)	<0.001
**Stage**						
Local	1.00^c^		1.00^c^		1.00^c^	
Regional	1.73 (1.69,1.77)	<0.001	1.12 (1.06,1.18)	<0.001	4.41 (4.21,4.62)	<0.001
Distant	10.39 (10.09,10.71)	<0.001	0.57 (0.50,0.65)	<0.001	27.57 (26.25,28.95)	<0.001
Unknown	1.50 (1.41,1.60)	<0.001	0.94 (0.81,1.09)	0.41	3.57 (3.22,3.97)	<0.001
**Treatment**						
RP	1.00^c^		1.00^c^		1.00^c^	
RT	2.64 (2.58,2.70)	<0.001	2.22 (2.13,2.32)	<0.001	4.30 (4.10,4.52)	<0.001

a *Representing multivariable Cox proportional hazards regression, adjusted for age at diagnosis, year of diagnosis, race, histologic subtype, grading, stage and treatment*.

b *Representing multivariable-adjusted competing risks, adjusted for age at diagnosis, year of diagnosis, race, histologic subtype, grading, stage, and treatment*.

c *The reference*.

*ADENO-CA, adenocarcinoma; HR, hazard ratio; SHR, subdistribution hazard ratio*.

Compared with Caucasians, African-American patients had a higher risk of heart-specific death (SHR, 1.42; 95% CI, 1.37–1.48), while patients of other ethnicities had a lower risk (SHR, 0.73; 95% CI, 0.68–0.79) (Table 3).

The risk of heart-specific death in patients with histologic subtypes other than adenocarcinoma was the same as that for adenocarcinoma (Table 3). Compared with patients with local disease, those with regional showed a significantly higher risk of heart-specific death (SHR, 1.21; 95% CI, 1.06–1.18). However, the risk of heart-related death in patients with distant disease was significantly less relative to local disease (SHR, 0.57; 95% CI, 0.50–0.65). Compared with patients who underwent RP, those who received RT had a significantly higher risk of heart-specific death (SHR 2.22; 95% CI, 2.13–2.32).

The benefits of RP and RT treatment with regard to heart-related mortality in patients with various PCa stages were further analyzed according to age group (Table 4). For patients older than 50 years given RP, those with distant disease had significantly lower rates of heart-related mortality than did those with local disease. However, patients with regional disease aged 50–79 years and given RT showed higher risk of heart-related mortality relative to those with local disease.

**Table 4:** Heart-related mortality in patients with PCa of various cancer stages who underwent RP or RT at diagnosis, analyzed by age and treatment subgroup.

		**Age** ** <50**		**Age 50–64**		**Age 65–74**		**Age** **≥75**	
	**Stage**	**SHR^**a**^**	***P***	**SHR^**a**^**	***P***	**SHR^**a**^**	***P***	**SHR^**a**^**	***P***
All	Local	1.00^b^		1.00^b^		1.00^b^		1.00^b^	
	Regional	1.07 (0.69–1.64)	0.756	1.21 (1.10,1.32)	<0.001	1.08 (0.99,1.16)	0.071	1.02 (0.90,1.15)	0.807
	Distant	0.86 (0.42–1.76)	0.683	0.71 (0.54,0.93)	0.012	0.61 (0.49,0.76)	<0.001	0.49 (0.40,0.59)	<0.001
	Unknown	0.91 (0.3–2.54)	0.865	0.98 (0.67,1.44)	0.919	0.95 (0.75,1.20)	0.67	0.91 (0.72,1.16)	0.192
RP	Local	1.00^b^		1.00^b^		1.00^b^		1.00^b^	
	Regional	0.72 (0.17–3.03)	0.652	0.94 (0.75–1.17)	0.568	0.99 (0.87–1.15)	0.986	1.02 (0.89–1.18)	0.736
	Distant	0.22 (0.03–1.71)	0.146	0.68 (0.51–0.89)	0.006	0.59 (0.47–0.74)	<0.001	0.48 (0.39–0.59)	<0.001
	Unknown	0.00 (0.00–0.00)	<0.001	0.96 (0.63–1.46)	0.837	0.96 (0.75–1.23)	0.762	0.95 (0.75–1.2)	0.679
RT	Local	—		1.00^b^		1.00^b^		1.00^b^	
	Regional	—		1.28 (1.16–1.42)	<0.001	1.13 (1.03–1.25)	0.014	1.08 (0.84–1.39)	0.554
	Distant	—		0.35 (0.05–2.45)	0.289	0.75 (0.18–3.03)	0.683	0.76 (0.18–3.14)	0.707
	Unknown	—		0.96 (0.36–2.55)	0.93	0.81 (0.32–2.06)	0.665	0.00 (0.00–0.00)	<0.001

a *Representing multivariable-adjusted competing risks*.

b *The reference*.

*HR, hazard ratio; SHR, subdistribution hazard ratio*.

## Discussion

This large population-based study analyzed the long-term heart-specific mortality of patients with PCa who had been treated with RT or RP. It was found that the rates of death of patients due specifically to heart-related disease increased steadily with age, and more so in patients receiving RT. The long-term heart-specific mortality of the patients overall was comparable to that of the general male population, but not for men 70–79 years of age who had received RT, for whom it was higher. These results highlight the value of incorporating risk stratification and pretreatment screening into the routine care of patients with PCa, to lower the risk of cardiovascular disease and improve patient survival.

Over the past few decades, PCa-specific mortality has gradually decreased, while more patients die from causes other than PCa, especially diseases that are heart related (2, 17, 18). Age- and treatment-induced complications may be important factors that increase the risk of heart-specific death (12, 19). The present study found that with increasing age, the risk of heart-specific deaths in patients with PCa gradually increased. In particular, about 9 years after the diagnosis of patients receiving RT, the cumulative mortality of heart-specific diseases exceeded that due to PCa. This association was not observed in patients treated with RP. These results did not acknowledge differences in baseline characteristics such as age at diagnosis, cancer stage, or tumor grade. More importantly, patients receiving RT were more likely to die from PCa and heart disease, which is consistent with previous results (20).

To minimize the influence of age, the overall patient cohort was compared with the general male population. After the age adjustment, the long-term heart-specific mortality of patients receiving RT or RP was slightly lower or comparable to that of the general population.

However, some studies have found that patients given RT had higher rates of cardiovascular disease (21, 22). A Canadian study investigated the link between different treatments and causes of cardiovascular death. The study reported that among patients who received RT, the 10-year cumulative incidence of non-PCa mortality was higher than that of patients who underwent surgery (21). The possible reason is that previous studies did not consider the reliable age-associated rise in cumulative heart-specific mortality in the general population. Although studies have shown that RT may induce cardiovascular disease via fibrosis, inflammatory infiltration, and hypertension caused by RT nephropathy, these diseases do not necessarily lead to death (23–25). In addition, the age of patients with PCa who receive RT tends to be higher than that of patients who undergo RP, and therefore, the basic condition of the body is worse. This makes the interpretation of analyses more challenging.

Interestingly, the present study found that for patients aged <65 years and given RT, the cumulative PCa mortality was higher than the cumulative heart-specific mortality, while for those treated with RP, the cumulative deaths due to heart disease and PCa were similar. In patients aged ≥65 years, given RT or RP, at ~7.5 years after diagnosis, the cumulative heart-specific mortality exceeded that of PCa. This may be because age is an independent risk factor for the occurrence and death from cardiovascular disease. The difference in baseline characteristics of patients with different treatments and potential treatment-induced complications may also have a certain influence (12, 19).

In addition, among patients receiving RT in the present study, the long-term heart-specific mortality of men aged 70–79 years was higher than that of the general population. This is consistent with previous results; the condition of these men is generally lower than that of men receiving RP (20). In addition, the interpretation of current SMRs is not easy because risk factors other than gender, age, and stage may compromise these results.

Based on the objective of this analysis, we used the Fine–Gray model of competitive risk to examine the potential prognostic factors for heart-specific mortality. This study found that the risk of heart death gradually increases with age, which confirms that age is a natural driver of cardiovascular disease (12). Compared with Caucasians, African-American patients have a higher risk of heart-specific death, while patients of other ethnicities have a lower risk of death. Some studies have also found that African-American patients are more susceptible to metastatic disease than other ethnicities, and the incidence of PCa, PCa-specific mortality, and all-cause mortality are higher (26–28).

The effect of age and stage on heart-specific mortality associated with RT or RP was further analyzed. For the subgroup aged 50–74 years treated with RT, patients with regional PCa has a higher heart-specific death rate compared with those with local disease, while no difference was found between local and distant PCa. However, patients with distant PCa who underwent RP had a lower risk of heart-specific death compared with local, while that of local and regional disease was similar. We consider that patients with higher-stage PCa may die from the cardiotoxic effects of treatment before the development of long-term heart-specific disease (29, 30). Therefore, when selecting a specific treatment, it is important to consider the susceptibility to cardiotoxic effects conferred by the patient's age and stage.

This study has some limitations. First, the type, dose, and duration of RT are unclear, and there may be differences between RT and RP selection criteria. Second, in some cases, the cause of heart death may not have been reported, and other causes of death may be unregistered. Finally, there is no data in SEER regarding some complicating risk factors of heart disease, such as diet, physical condition, and geographical environment, which may influence the results.

In summary, this study identified some potential prognostic factors for heart and PCa-specific mortality. More importantly, the study showed that the heart-specific mortality of patients with PCa was similar to that of the general male population, and therefore, RT or RP treatments for PCa did not increase the risk of death due to heart disease. However, among the men receiving RT for PCa, the long-term heart-specific mortality of those aged 70–74 years was higher than that of the general male population. This may provide a basis for risk stratification of heart disease in patients with PCa and help clinicians determine the appropriate cardiovascular disease screening strategies and interventions for this population.

## Data Availability Statement

All datasets generated for this study are included in the article/Supplementary Material.

## Author Contributions

Conceptualization of the study was by YG and XY. Data curation was performed by XD and FY. YY and FY did the formal analysis of the study. Funding acquisition was done by XY. RW and SM contributed to the investigation. AZ contributed to the methodology. AK and XY provided the resources. Software support was done by AZ. WZ and AZ provided supervision, whereas XD was responsible for validation. Visualization was performed by WZ and YY. The original draft was written by YG and reviewed and edited by XY. All authors interpreted the data, edited or commented, and approved the final manuscript.

## Conflict of Interest

The authors declare that the research was conducted in the absence of any commercial or financial relationships that could be construed as a potential conflict of interest.
