# Supplementary material for: Effects of Radiotherapy or Radical Prostatectomy on the Risk of Long-Term Heart-Specific Death in Patients With Prostate Cancer
Source: Front Oncol. 2020 Nov 17;10:592746. doi: 10.3389/fonc.2020.592746 (PMC7720700; doi:10.3389/fonc.2020.592746)
Supplement: Supplementary file 1 [file Table_1.DOCX]

**Supplementary Table 1.** Definition of each cause of death and corresponding codes in the ICD-10 of Diseases and Related Health.

| Cause of death | ICD-10 corresponding codes | Cause of death definition |
| --- | --- | --- |
| Diseases of Heart | I00-I02 | Acute rheumatic fever |
|  | I05-I09 | Chronic rheumatic heart diseases |
|  | I11 | Hypertensive heart disease |
|  | I13 | Hypertensive heart and renal disease |
|  | I20-I25 | Ischemic heart diseases |
|  | I26-I28 | Pulmonary heart disease and diseases of pulmonary circulation |
|  | I30-I32 | Diseases of pericardium |
|  | I33 | Acute and subacute endocarditis |
|  | I34-I39 | Nonrheumatic valve disorders |
|  | I40-I41 | Myocarditis |
|  | I42-I43 | Cardiomyopathy |
|  | I44-I45 | Conduction disorders |
|  | I46 | Cardiac arrest |
|  | I47-I49 | Arrythmias |
|  | I50 | Heart failure |
|  | I51 | Complications and ill-defined descriptions of heart disease |

**Supplementary Table 2**. All-cause, heart-related, and prostate-related hazard ratio of patients adjusted for demographic and clinical characteristics at diagnosis in patients surviving at least 10 years.

| variables | all cause | |  | heart diseases | |  | prostate | |
| --- | --- | --- | --- | --- | --- | --- | --- | --- |
|  | HR | P value |  | sHR | P value |  | sHR | P value |
| Age |  |  |  |  |  |  |  |  |
| <50 | 0.50(0.42,0.60) | <0.001 |  | 0.44(0.30,0.64) | <0.001 |  | 0.61(0.44,0.85) | 0.003 |
| 50-64 | 1.00c |  |  | 1.00c |  |  | 1.00c |  |
| 65-74 | 2.57(2.46,2.69) | <0.001 |  | 2.70(2.47,2.95) | <0.001 |  | 1.17(1.05,1.29) | 0.003 |
| ≥75 | 5.99(5.69,6.31) | <0.001 |  | 6.59(5.94,7.30) | <0.001 |  | 1.39(1.20,1.61) | <0.001 |
| Year of diagnosis |  |  |  |  |  |  |  |  |
| 2000–2005 | 1.00 (ref.) |  |  | 1.00 (ref.) |  |  | 1.00 (ref.) |  |
| 2006–2012 | 0.79(0.78-0.81) | <0.001 |  | 0.67(0.65,0.70) | <0.001 |  | 0.63(0.61,0.65) | <0.001 |
| Race |  |  |  |  |  |  |  |  |
| White | 1.00c |  |  | 1.00c |  |  | 1.00c |  |
| Black | 1.21(1.15,1.27) | <0.001 |  | 1.27(1.15,1.40) | <0.001 |  | 1.03(0.90,1.18) | 0.642 |
| Other | 0.81(0.75,0.88) | <0.001 |  | 0.76(0.65,0.89) | 0.001 |  | 0.76(0.61,0.95) | 0.015 |
| Unknown | 0.24(0.16,0.37) | <0.001 |  |  |  |  | 0.29(0.11,0.77) | 0.013 |
| Histologic subtype |  |  |  |  |  |  |  |  |
| Adenocarcinoma | 1.00c |  |  | 1.00c |  |  | 1.00c |  |
| Other | 0.90(0.81,1.00) | 0.046 |  | 0.78(0.62,0.98) | 0.03 |  | 0.80(0.61,1.04) | 0.097 |
| Grading |  |  |  |  |  |  |  |  |
| I | 1.00c |  |  | 1.00c |  |  | 1.00c |  |
| II | 0.99(0.88,1.11) | 0.822 |  | 0.96(0.77,1.19) | 0.702 |  | 1.46(0.94,2.27) | 0.962 |
| III | 1.29(1.14,1.45) | <0.001 |  | 1.10(0.87,1.38) | 0.418 |  | 3.78(2.43,5.90) | <0.001 |
| IV | 1.57(1.10,2.25) | 0.012 |  | 0.97(0.42,2.21) | 0.935 |  | 5.69(2.70,11.99) | <0.001 |
| Unknown | 1.28(1.09,1.50) | 0.002 |  | 1.03(0.76,1.41) | 0.83 |  | 3.06(1.86,5.02) | <0.001 |
| Stage |  |  |  |  |  |  |  |  |
| Local | 1.00c |  |  | 1.00c |  |  | 1.00c |  |
| Regional | 1.47(1.39,1.55) | <0.001 |  | 1.15(1.03,1.29) | 0.015 |  | 3.22(2.86,3.62) | <0.001 |
| Distant | 1.89(1.52,2.36) | <0.001 |  | 0.94(0.53,1.66) | 0.826 |  | 6.86(5.10,9.23) | <0.001 |
| Unknown | 1.22(1.04,1.42) | 0.013 |  | 0.78(0.54,1.12) | 0.184 |  | 3.39(2.60,4.42) | <0.001 |
| Treatment |  |  |  |  |  |  |  |  |
| RP | 1.00c |  |  | 1.00c |  |  | 1.00c |  |
| RT | 2.00(1.91,2.09) | <0.001 |  | 2.05(1.87,2.25) | <0.001 |  | 3.25(2.89,3.66) | <0.001 |

**Supplementary Table 3**. Heart-related mortality in PCa who survived at least 10 years of various cancer stages who underwent RP or RT at diagnosis, analyzed by age and treatment subgroup.

|  |  | Age<50 | |  | Age 50-64 | |  | Age 65-74 | |  | Age≥75 | |
| --- | --- | --- | --- | --- | --- | --- | --- | --- | --- | --- | --- | --- |
|  |  | sHR | P value |  | sHR | P value |  | sHR | P value |  | sHR | P value |
| All | Stage |  |  |  |  |  |  |  |  |  |  |  |
|  | Local | 1.00 (ref.) |  |  | 1.00 (ref.) |  |  | 1.00 (ref.) |  |  | 1.00 (ref.) |  |
|  | Regional | 1.51(0.60,3.80) | 0.386 |  | 1.39(1.14,1.70) | 0.001 |  | 1.02(0.87,1.20) | 0.816 |  | 1.07(0.78,1.45) | 0.675 |
|  | Distant | (.) |  |  | 1.49(0.56,4.00) | 0.429 |  | 0.68(0.25,1.83) | 0.44 |  | 0.97(0.36,2.59) | 0.953 |
|  | Unknown | (.) |  |  | 0.81(0.34,1.91) | 0.626 |  | 0.83(0.49,1.40) | 0.484 |  | 0.72(0.38,1.37) | 0.313 |
| RP | Stage |  |  |  |  |  |  |  |  |  |  |  |
|  | Local | (.) |  |  | 1.00 (ref.) |  |  | 1.00 (ref.) |  |  | 1.00 (ref.) |  |
|  | Regional | (.) |  |  | 1.21(0.76,1.94) | 0.418 |  | 0.87(0.62,1.22) | 0.421 |  | 1.21(0.85,1.71) | 0.294 |
|  | Distant | (.) |  |  | 1.22(0.39,3.76) | 0.733 |  | 0.72(0.26,1.94) | 0.512 |  | 1.03(0.39,2.76) | 0.947 |
|  | Unknown | (.) |  |  | 0.90(0.38,2.15) | 0.816 |  | 0.90(0.52,1.54) | 0.692 |  | 0.75(0.40,1.43) | 0.388 |
| RT | Stage |  |  |  |  |  |  |  |  |  |  |  |
|  | Local | (.) |  |  | 1.00 (ref.) |  |  | 1.00 (ref.) |  |  | 1.00 (ref.) |  |
|  | Regional | (.) |  |  | 1.42(1.13,1.77) | 0.002 |  | 1.08(0.89,1.31) | 0.435 |  | 0.85(0.50,1.43) | 0.535 |
|  | Distant | (.) |  |  | 2.77(0.38,20.05) | 0.312 |  | 0.00(0.00,0.00) | <0.001 |  | 0.00(0.00,0.00) | <0.001 |
|  | Unknown | (.) |  |  | 0.00(0.00,0.00) | <0.001 |  | 0.50(0.07,3.36) | 0.473 |  | 0.00(0.00-0.00) | <0.001 |

| **Supplementary Table 4.** Age-specific heart diseases SMRs for the years 2012 to 2014 among patients with Pca relative to the general male population of the United States by race* | | | | | |
| --- | --- | --- | --- | --- | --- |
| Ethnicities | Age | All patients with Pca | | Patients with Pca survived at least 10years | |
|  |  | RT | RP | RT | RP |
| Caucasian | 50-54 | 1.27(0.92,1.75) | 0.41(0.31,0.54) |  | 1.13(0.28,4.52) |
|  | 55-59 | 1.03(0.87,1.21) | 0.41(0.35,0.48) |  | 0.73(0.38,1.4) |
|  | 60-64 | 1.01(0.92,1.11) | 0.38(0.34,0.42) | 1.26(0.8,1.98) | 0.27(0.16,0.44) |
|  | 65-69 | 0.94(0.88,1) | 0.39(0.36,0.42) | 1.06(0.82,1.38) | 0.45(0.36,0.57) |
|  | 70-74 | 0.94(0.9,0.99) | 0.44(0.41,0.48) | 1.14(0.97,1.33) | 0.5(0.42,0.59) |
|  | 75-79 | 0.89(0.86,0.93) | 0.49(0.45,0.53) | 1.09(0.98,1.21) | 0.53(0.46,0.61) |
|  | 80-85 | 0.9(0.86,0.93) | 0.54(0.49,0.6) | 0.99(0.91,1.07) | 0.59(0.52,0.68) |
|  | 85+ | 0.75(0.72,0.78) | 0.49(0.42,0.58) | 0.9(0.85,0.96) | 0.51(0.42,0.62) |
|  | subtotal | **0.87(0.86,0.89)** | **0.44(0.42,0.46)** | 0.97(0.93,1.01) | **0.52(0.48,0.56)** |
| Aafrican-American | 50-54 | 1(0.67,1.49) | 0.66(0.47,0.93) |  |  |
|  | 55-59 | 1.22(1.01,1.47) | 0.47(0.37,0.6) | 1.03(0.26,4.13) | 0.53(0.17,1.63) |
|  | 60-64 | 1.01(0.89,1.16) | 0.71(0.61,0.82) | 0.82(0.41,1.64) | 0.86(0.53,1.38) |
|  | 65-69 | 1(0.9,1.11) | 0.54(0.47,0.63) | 0.92(0.61,1.4) | 0.59(0.39,0.89) |
|  | 70-74 | 1.01(0.92,1.1) | 0.52(0.44,0.61) | 1.18(0.9,1.54) | 0.6(0.42,0.86) |
|  | 75-79 | 0.88(0.8,0.96) | 0.64(0.53,0.77) | 0.95(0.75,1.21) | 0.72(0.53,0.97) |
|  | 80-85 | 0.85(0.76,0.95) | 0.79(0.59,1.05) | 0.89(0.71,1.12) | 0.82(0.56,1.19) |
|  | 85+ | 1.02(0.9,1.16) | 0.93(0.55,1.56) | 1.15(0.94,1.4) | 1.03(0.57,1.86) |
|  | subtotal | 0.96(0.92,1) | **0.6(0.56,0.64)** | 1.01(0.91,1.13) | **0.7(0.6,0.82)** |
| other/unknown ehnicities | 50-54 | 2.27(0.73,7.04) | 1.35(0.56,3.24) |  |  |
|  | 55-59 | 0.72(0.27,1.91) | 0.57(0.27,1.2) | 11.2(1.58,79.51) |  |
|  | 60-64 | 1.26(0.83,1.92) | 0.26(0.13,0.52) | 1.79(0.25,12.69) | 0.72(0.1,5.09) |
|  | 65-69 | 1(0.73,1.37) | 0.45(0.29,0.68) | 1.04(0.26,4.17) |  |
|  | 70-74 | 0.92(0.74,1.16) | 0.3(0.19,0.47) | 1.43(0.68,3.01) | 0.13(0.02,0.92) |
|  | 75-79 | 0.96(0.81,1.14) | 0.61(0.43,0.85) | 1.53(0.99,2.37) | 0.64(0.32,1.29) |
|  | 80-85 | 0.9(0.77,1.06) | 0.64(0.41,1) | 1.16(0.82,1.63) | 0.79(0.42,1.46) |
|  | 85+ | 0.87(0.75,1) | 1.15(0.7,1.91) | 1.07(0.85,1.35) | 0.73(0.33,1.62) |
|  | subtotal | 0.92(0.85,1) | **0.5(0.42,0.59)** | 1.17(0.99,1.39) | **0.55(0.38,0.81)** |
